# Quantitative Equivalence and Performance Comparison of Particle and Field-Theoretic Simulations

**DOI:** 10.1021/acs.macromol.4c02034

**Published:** 2024-11-12

**Authors:** Joshua Lequieu

**Affiliations:** Department of Chemical and Biological Engineering, Drexel University, Philadelphia, Pennsylvania 19104, United States

## Abstract

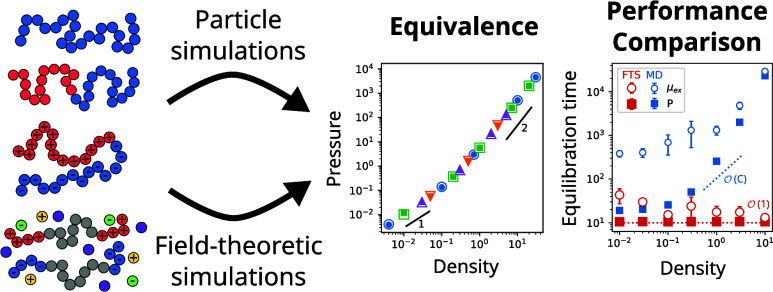

Particle and field-theoretic simulations are both commonly used methods to study the equilibrium properties of polymeric materials. Yet despite the formal equivalence of the two methods, no comprehensive comparisons of particle and field-theoretic simulations exist in the literature. In this work, we seek to fill this gap by performing a systematic and quantitative comparison of particle and field-theoretic simulations. In our comparison, we consider four representative polymeric systems: a homopolymer melt/solution, a diblock copolymer melt, a polyampholyte solution, and a polyelectrolyte gel. For each of these systems, we first demonstrate that particle and field-theoretic simulations are equivalent and yield exactly the same results for the pressure and the chemical potential. We next quantify the performance of each method across a range of different conditions including variations in chain length, system density, interaction strength, system size, and polymer volume fraction. The outcome of these calculations is a comprehensive look into the performance of each method and the systems and conditions when either particle or field-theoretic simulations are preferred. We find that field-theoretic simulations are equal to or faster than particle simulations for nearly all of the systems and conditions examined. In many situations, field-theoretic simulations are several orders of magnitude faster than particle simulations, especially if the polymer chains are long, the system density is high, and long-range Coulombic interactions are present. We also demonstrate that field-theoretic simulations are considerably faster at calculating the chemical potential and bypass the challenges associated with particle-based Widom insertion techniques. Taken together, our results provide quantitative evidence that field-theoretic simulations can reach and sample equilibrium considerably faster than particle simulations while simultaneously producing equivalent results.

## Introduction

Simulations of polymers can generally be classified into either particle-based or field-based methods.^1,2^ When equilibrium properties are of interest, the primary goal of both particle- and field-based simulations is to sample a partition function. In a particle-based method, the canonical partition function for a system with *n* molecules at volume *V* and temperature *T* is given by
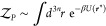
1where *r*^3*n*^ denotes the positions of all particles, β = 1/*k*_*B*_*T*, and *U*(***r***^*n*^) is the potential energy.^3^ The objective of particle-based simulations, such as molecular dynamics (MD) or Monte Carlo, is to numerically construct a trajectory ***r***^*n*^(*t*) that samples particle coordinates according to the probability *P*(***r***^*n*^) ∼ exp(−*βU*(***r***^*n*^)).^4,5^ Once this trajectory is obtained, it can be used to compute both structural and thermodynamic information about a system. The accuracy of particle-based simulations depends on whether the probability distribution is adequately sampled by ***r***^*n*^(*t*) and on the fidelity of the underlying numerical methods used to generate this trajectory. If these errors are mitigated, then particle-based simulations are an exact solution to the underlying physical model defined by *U*(***r***^*n*^).

In a field-based simulation, the canonical partition function is instead given by
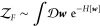
2where ***w*** = {*w*_1_, *w*_2_, ···} is a set of fields and *H*[***w***] is a field-based Hamiltonian that is a functional of ***w***. While it is common to approximate eq 2 using mean-field methods such as self-consistent field theory (SCFT), it is also possible to sample eq 2 without approximation using field-theoretic simulations (FTS).^6−8^ In direct analogy to particle-based simulations, the objective of field-theoretic simulations is to numerically construct a trajectory ***w***(*t*) that samples field configurations according to the probability *P*(***w***) ∼ exp(−*H*[***w***]). One complication when constructing ***w***(*t*) is that *H*[***w***] is often complex-valued, which results in nonpositive-definite weights (i.e., a sign problem) that necessitate specialized numerical techniques.^7,8^ Once the trajectory ***w***(*t*) is obtained, it can be used to compute a variety of structural and thermodynamic quantities of a system, and its accuracy depends on both sufficient sampling and accurate numerical methods. If sampling and numeric errors are avoided, field-theoretic simulations also represent an exact solution to the underlying physical model.

From this brief discussion, it is evident that particle and field-theoretic simulations are conceptually quite similar and that both methods seek to numerically sample the partition functions  and , respectively. For certain restrictions on the functional form of *U*(***r***^*n*^), it can be to shown that particle and field-based partition functions are formally equivalent and that .^7,8^ These restrictions require that interactions between particles are pairwise additive and that the pair interaction potentials be both positive-definite and have a functional inverse.^7,8^ If these restrictions are satisfied, then the equality of  and  indicates that both particle and field-theoretic simulations are also formally equivalent and that the methods should yield equal results. Thus, in a mathematically rigorous sense, particle and field-theoretic simulations represent two independent routes to obtain the same information about a system.

This equivalence has several important implications for the simulation of polymers. One implication is that field-theoretic simulations can enable the study of polymeric systems that might otherwise be intractable with particle simulations alone. For example, while particle simulations become more expensive as a system’s density increases, field-theoretic simulations are generally thought to become more efficient in this regime. As a consequence, field-theoretic simulations can accelerate calculations of dense polymeric systems and should produce the same results as particle simulations since . Another implication is that new simulation methods can be designed that leverage the equivalence of the two methods. For example, Fredrickson and co-workers have used this equivalence to examine polyampholyte coacervation using both particle and field-theoretic simulations^9−11^ and have recently extended this approach to devise molecularly informed field theories whose parameters are obtained from atomistic particle-based simulations.^12−16^ In other recent work, Riggleman and co-workers have embedded this equivalence into their recently developed MATILDA.FT simulation package^17^ and the associated theoretically informed Langevin dynamics method.^18−20^ This equivalence has also between exploited by our group to develop multi-representation simulations where combined particle and field-theoretic simulations are converted between one another in order to accelerate equilibration and free energy calculations while also maintaining information about chain configurations and dynamics.^21^

While these methods can be quite powerful, their utility relies on a quantitative understanding of the conditions when either particle or field-theoretic simulations are most efficient. Unfortunately, such a quantitative understanding is almost completely absent from the literature. For instance, while it has been argued for decades that field-theoretic simulations are generally preferred when the density is high or polymers are long,^7^ quantitative evidence to support these claims has only appeared within the past year.^8,21^ Moreover, since these recent comparisons have only been performed for simple systems (e.g., homopolymers in implicit solvent), it is unclear whether they can be generalized to systems of higher complexity.

Another open question is whether particle and field-theoretic simulations actually yield equivalent results in practice. In principle, particle and field-theoretic should be equivalent since , but in practice this equivalence will only be achieved if numerical and sampling errors are eliminated in both methods. The elimination of these errors can be challenging and should not be assumed, especially in field-theoretic simulations, which are considerably less mature than particle simulations. Unfortunately, past work that has directly compared particle and field-theoretic simulations is quite limited. In instances where the predictions of these methods have been directly compared, the comparisons were cursory (e.g., a single panel of a figure) and were limited to relatively simple observables like the pressure.^9,18,21^ More systematic studies are needed to establish the conditions where particle- and field-theoretic simulations are equivalent and the necessary numeric or sampling requirements for both methods to yield equal results.

The goal of this Article is to address these gaps and to provide a systematic and comprehensive comparison of particle and field-theoretic simulations. By comparing these two methods across a range of systems and conditions, our work seeks both to evaluate when particle and field-theoretic simulations provide equivalent results and to quantify the performance of each method. To achieve this goal, we first develop a generalized polymer model that can be exactly simulated using both particle and field-theoretic simulations. This model incorporates both short-range and long-range (i.e., Coulombic) interactions and is sufficiently general so as to represent any number of sequence-defined polymers consisting of any number of species. Using this generalized model, we then perform a systematic comparison of particle and field-theoretic simulations for four systems of increasing complexity: (1) a homopolymer solution or melt, (2) a diblock copolymer melt, (3) polyampholytes in implicit solvent, and (4) polyelectrolyte gels with explicit solvent and salt ions. For each system, we first establish the quantitative equivalence of particle and field-theoretic simulations by demonstrating that the two methods yield identical results for both the pressure and the chemical potential. We next quantify the computational cost of both particle and field-theoretic simulations and how their performance depends on a myriad of parameters such as the total density, polymer chain length, system size, polymer volume fraction, and electrostatic strength. Since our methodology is consistent across these different systems and conditions, our work can provide a comprehensive picture of the situations where either particle or field-theoretic simulations are preferred. We envision that these results will have implications both for field-theoretic simulations and for emerging methods that seek to combine them with particle simulations.

## Methods

In order to provide a systematic comparison between particle and field-theoretic simulations, we first formulate a model that can be simulated using both methods. The model that we have chosen is quite general and is sufficiently flexible to examine a wide range of different systems encompassing polymer solutions and melts with both short- and long-range Coulombic interactions. This general model enables the direct comparison of particle and field-theoretic simulations and can be used to provide a comprehensive picture of when each method is most efficient. We note that this model has been previously studied using the random-phase approximation,^13^ self-consistent field theory,^14,16^ and molecular dynamics simulations^13,14,16,22^ but not with field-theoretic simulations.

### Particle-Based Representation

The model we consider consists of *n* total molecules with *M* different types at temperature *T* in volume *V* consisting of an orthorhombic box with dimensions *L*_*x*_, *L*_*y*_, and *L*_*z*_. Each molecule of type *m* has *n*_*m*_ indistinguishable copies such that *n* = ∑_*m*_^*M*^*n*_*m*_. Each molecule consists of a linear polymer of covalently bonded beads with a degree of polymerization *N*_*m*_. Small molecules or salt ions can be represented by choosing *N*_*m*_ = 1. For compactness, *n*_*m*_ and *N*_*m*_ for each molecule type are represented by the vectors ***n*** = {*n*_1_, *n*_2_, ···, *n*_*M*_} and ***N*** = {*N*_1_, *N*_2_, ···, *N*_*M*_} so that the total number of beads in the system is given by ***n*** · ***N*** = ∑_*m*_^*M*^*n*_*m*_*N*_*m*_ and the overall number density is given by ρ_0_ = ***n***·***N***/*V*. The volume fraction of each molecule is given by ϕ̅_*m*_ = *n*_*m*_*N*_*m*_/***n***·***N***.

Each bead type *i* is chosen from the total number of *S* species present in the system and is assigned charge *z*_*i*_, characteristic size *a*_*i*_, and statistical segment length *b*_*i*_. Beads of type *i* and *j* separated by distance *r* interact via the nonbonded pair-potential
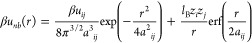
3where the first term represents excluded volume interactions, the second term represents regularized Coulombic interactions, *l*_B_ is the Bjerrum length, and *a*_*ij*_ = ((*a*_*i*_^2^ + *a*_*j*_^2^)/2)^1/2^. The strength of the excluded volume interactions is given by *βu*_*ij*_ = *βu*_0_ + χ_*ij*_/ρ_0_ where χ_*ij*_ is the Flory interaction parameter and *u*_0_ > 0 controls the overall compressibility of the system. Bonded beads interact via the potential
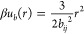
4where *b*_*ij*_ = (*b*_*i*_*b*_*j*_)^1/2^ is the characteristic length of a bond between beads types *i* and *j*. As we will demonstrate shortly, this model is sufficiently general to describe a broad range of polymeric systems ranging from solutions to melts involving any combination of neutral and charged components.

The particle-based canonical partition function for this model is
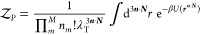
5where λ_T_ is the thermal de Broglie wavelength and *U*(***r***^***n***·***N***^) is the total potential energy given by
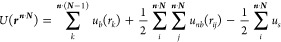
6where the first sum runs over all bonds and the second sum runs over all particle pairs, including self-interactions where *i* = *j*. The final term cancels the bead self-interactions included in the second nonbonded interaction term where *u*_*s*_ ≡ *u*_nb_(0) = *u*_0_/(8π^3/2^*a_i_*^3^) + *l*_B_*z*_*i*_^2^/(*βπ*^1/2^*a*_*i*_).

In order to sample this partition function and to examine the system’s dynamics, the particle positions are evolved using Langevin dynamics
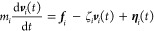
7where *m*_*i*_ is the mass of the *i*th particle, ***v***_*i*_(*t*) = d***r***_*i*_/d*t* is its velocity, ***r***_*i*_ is its position, ***f***_*i*_ = –*∂U*(***r***^***n***·***N***^)/∂***r***_*i*_ is the conservative force on the particle, and ζ_*i*_ is its friction coefficient. The final term **η**_*i*_ is Gaussian white noise with moments ⟨**η**_*i*_(*t*)⟩ = 0 and ⟨**η**_*i*_(*t*)·**η**_*i*_(*t′*)⟩ = 2ζ_*i*_*k*_B_*Tδ*(*t* – *t′*).

Coulombic interactions are solved using a particle–particle–particle–mesh (PPPM) scheme.^23,24^ Since the regularized Coulombic interactions in eq 6 differ slightly from the conventional Coulombic interactions (i.e., *u*_nb_ ∼ 1/*r*), some care must be taken to incorporate our model into existing PPPM implementations.^25^ We follow the strategy described by Kiss et al.^26^ and use a modified real-space potential
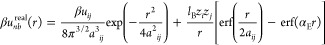
8where α_E_ is the Ewald parameter that tunes the relative weight of the real space and reciprocal space contributions.^24^ If eq 8 is used for the real-space potential, than conventional reciprocal space PPPM implementations can be used without modification to recover an overall nonbonded potential energy consistent with eq 6.^9,26^ We implement eq 8 using a tabulated potential with a discretization such that the absolute error between a pair of opposite charges is less than 10^–4^ for all separations *r*. Optimal grid resolutions and α_*E*_ are chosen for a given real-space cutoff *r*_*c*_ using the error estimates of ref (27). so that the error is also less than 10^–4^.

All simulations are performed in reduced units with *k*_B_*T* = 1, and all particles are assigned equal masses *m*_*i*_ = 1 and friction coefficients ζ_*i*_ = 2. The pairwise cutoff was set to be *r*_*c*_ = 6.4*a*_*ij*_ unless otherwise specified, and the time step was chosen as Δ*t* = 0.005τ where . A cell-based neighbor list was used to compute pairwise interactions.

### Field-Theoretic Representation

The particle-based model described in the previous section can be exactly converted to a field theory that can be simulated by using field-theoretic simulations. Specifically, our approach combines past work on a Gaussian regularization,^28−30^ multispecies exchange theories,^31^ and electrostatic interactions^7,32,33^ to convert the partition function in eq 5 into a field theory
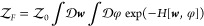
9where ***w*** = {*w*_1_, *w*_2_, ···, *w*_*R*_} contains *R* newly introduced auxiliary fields and φ is a field used to decouple the electrostatic interactions. *H*[***w***,φ] is a field-theoretic Hamiltonian given by
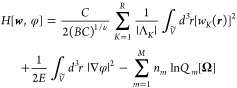
10where *B* = *βu*_0_*N*^2^/*R*_g_^3^ is a reduced excluded volume, *C* = ρ_0_*R*_g_^3^/*N* is a reduced chain number density, *E* = 4*πl*_B_*z*^2^*N*^2^/*R*_g_ is a reduced Bjerrum length, *Ṽ* = *V*/*R*_g_^3^ is a reduced volume, *z* is a reference charge, and *R*_g_ = *b*((*N* – 1)/6)^1/2^ is the unperturbed radius of gyration of a chain with degree of polymerization *N* and statistical segment length *b*. The coordinates *r* are expressed in units of *R*_g_, and factors of *N* and *zN* have been absorbed into the fields *w*_*K*_ and φ, respectively. The variables Λ_*K*_ correspond to the nonzero eigenvalues of the *S* × *S* matrix ***X*** = **χ***N*(*BC*)^−1/ν^ + (*BC*)^1–1/ν^**1**, where **χ** = (χ_*ij*_) with *i*, *j* ∈ [0, ···, *S*] and **1** is a matrix of ones. The rank of ***X*** (i.e., the number of nonzero eigenvalues) is given by *R*, and ν is an adjustable parameter that is chosen for numeric stability.

The prefactor  in eq 9 is given by
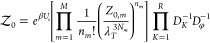
11where *U*_*s*_ is the total internal energy due to self-interactions
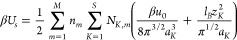
12and *N*_*K*,*m*_ is the number of beads of species *K* in molecule *m*. *Z*_0,*m*_ is the partition function of molecule *m* in the absence of nonbonded interactions
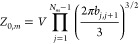
13and *D*_*K*_ and *D*_φ_ are the normalizing denominators from the Hubbard–Stratonovich transforms that introduced the *w*_*K*_ fields and the φ field, respectively.
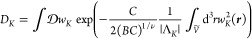
14
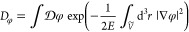
15

The final term to be specified in eq 10 is *Q*_*m*_[**Ω**], the single-chain partition function of molecule *m*, which is a functional of the fields **Ω** = {Ω_1_, Ω_2_, ···, Ω_*S*_} that are defined as
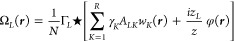
16for *L* = 1, ···, *S*. In this expression , ★ denotes a spatial convolution, Γ_*L*_ (*r*) = (2*πã*_*L*_^2^)^−3/2^exp[−*r*^2^/(2*ã*_*L*_^2^)], *ã*_*L*_ = *a*_*L*_/*R*_g_, and γ_*K*_ = *i* if Λ_*K*_ > 0 or γ_*K*_ = 1 otherwise. *A*_*LK*_ is an element of the *S* × *R* matrix ***A*** whose columns are the eigenvectors of ***X*** with nonzero eigenvalues. In situations where ***X*** has degenerate eigenvalues, some eigenvectors are not necessarily orthogonal, and a QR decomposition of these eigenvectors is required. *Q*_*m*_[**Ω**] is defined as
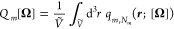
17where *q*_*m*,*j*_(***r***) is the propagator corresponding to the statistical weight for a molecule *m* at bead index *j* at position ***r*** for fields **Ω**. The propagator is computed from a Chapman–Kolmogorov equation
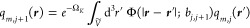
18where *K* is the species type of the *j* + 1 bead and *q*_*m*,1_(***r***) = exp(−Ω_*L*_(***r***)), where *L* is the species type of the first bead of molecule *m*. Φ(*r*) is the bond transition probability and is given by
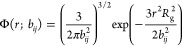
19for particles of type *i* and *j* connected by the bonded potential given in eq 4, and *r* is in units of *R*_g_. The conjugate propagator, *q*_*m*,*j*_^†^(***r***), is required to compute functional derivatives of *Q* and is obtained via
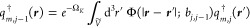
20where *K* is the species type of the *j* – 1 bead and , where *L* is the species type of the *N_m_*th bead of molecule *m*.

Due to the mathematical structure of this field theory, all observables must be computed in terms of the fields ***w*** and φ. One such observable is the local density of each species, ϕ̃_*K*_(***r***), which is computed as
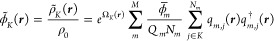
21where the sum over *j* only includes particles of type *K* and ϕ̃_*K*_(***r***) has been normalized by ρ_0_ so that it ranges from zero to approximately one. Other operators such as the pressure or chemical potential are discussed in the following section.

The field-based partition function in eq 9 is sampled using complex Langevin (CL) dynamics^34,35^ where the original real-valued ***w*** and φ fields are permitted to adopt complex values and are evolved in a fictitious time θ according to
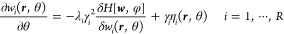
22
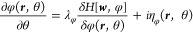
23where λ_*i*_, λ_φ_ > 0 are real-valued mobility coefficients and η_*i*_ is real-valued Gaussian white noise with moments ⟨η_*i*_(***r***, θ)⟩ = 0 and ⟨η_*i*_(***r***, θ)η_*i*_(***r****′*, θ′)⟩ = 2λ_*i*_δ(***r*** – ***r****′*)δ(θ – *θ′*). Equations 22 and 23 can also be used to locate mean-field configurations (i.e., SCFT) by setting η_*i*_ = η_φ_ = 0.

In order to perform field-theoretic simulations, all fields are discretized onto a uniform grid with *M*_*j*_ = ceil_2,3_(*L*_*j*_/a_min_) grid points in each dimension, where a_min_ is the smallest *a*_*i*_ in the system, ceil_2,3_(X) is a function that returns the smallest integer greater than X that can be represented by powers of 2 or 3, and *j* = *x*, *y*, *z*. The total number of grid points is *M*_*t*_ = *M*_*x*_*M*_*y*_*M*_*z*_. Equations 22 and 23 are integrated numerically using a Euler–Maruyama Predictor Corrector field updater^31,36^ with time step Δθ = 0.01–0.1. The stability of field-theoretic simulations is improved through tuning the mobility coefficients λ_*i*_, λ_φ_ for each system of interest for ν = 2. The propagators in eqs 18 and 20 and convolutions involving Γ_*K*_(***r***) are efficiently computed pseudospectrally using fast Fourier transforms.

### Observables in Particle and Field-Theoretic Simulations

In order to confirm that particle and field-theoretic simulations yield equivalent results, we next needed to derive expressions for different thermodynamic observables that can be computed using both methods. While there are numerous observables that could be used to compare the two methods, we choose to restrict our focus to two observables: the pressure and the chemical potential.

#### Pressure in Particle and Field-Theoretic Simulations

The first observable we focus on is the pressure, which is defined thermodynamically as *P* ≡ −(*∂A*/*∂V*)_***n***,*T*_, where *A* is the Helmholtz free energy and . For a particle-based model, eq 5 can be differentiated with respect to *V* to obtain the well-known virial expression for the pressure^3^
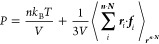
24where the first term is the ideal gas contribution and ⟨··· ⟩_***r***^***n*** ·***N***^_ denotes an ensemble average over particle configurations.

For a field-theoretic model, we instead differentiate eq 9 with respect to *V* to obtain an expression for the pressure. We compute this derivative using a volume-rescaling approach^8,30^ to obtain
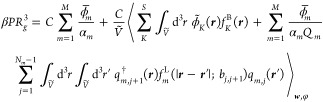
25where the first term is the ideal gas contribution, the second term results from nonbonded interactions, the final term results from bonded interactions, and ⟨··· ⟩_***w***,φ_ denotes an ensemble average over field configurations. In this expression, α_*m*_ = *N*_*m*_/*N* is a normalized chain length, *f*_*K*_^B^(***r***) is a bead function for species type *K*, and *f*_*m*_^L^(*r*;*b*_*ij*_) is a link function for bonded beads *i* and *j* in molecule *m*. These functions are equal to
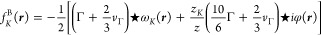
26
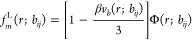
27where
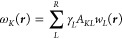
28and *v*_Γ_(*r*) and *v*_*b*_(*r*) are virial functions.
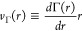
29
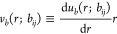
30

Despite the very different forms of eqs 24 and 25, these two equations are formally equivalent expressions for computing the pressure. We will demonstrate this equivalence in subsequent sections of this Article. We will also analyze whether the pressure computed via a particle-based simulation in eq 24 or a field-theoretic simulation in eq 25 converges to its equilibrium value more quickly.

#### Chemical Potential in Particle and Field-Theoretic Simulations

We also seek to compute the chemical potential μ_*m*_, which is defined thermodynamically as μ_*m*_ ≡ (*∂A*/*∂n*_*m*_)_*n*_*j*≠*m*_,*T*_. In particle-based simulations, *n*_*m*_ appears in the integration measure of eq 5 and so this derivative cannot be taken directly. Instead, this derivative is approximated by a finite difference between a system containing *n*_*m*_ + 1 and *n*_*m*_ copies of molecule *m* and results in the well-known Widom insertion method for the excess chemical potential^4,5^
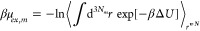
31where the integral spans all possible configurations of the *N*_*m*_ beads in molecule *m*, Δ*U* = Δ*U*_b_ + Δ*U*_nb_ is the total change in the bonded and nonbonded internal energy due to the inserted molecule, and ⟨··· ⟩_*r*^***n***·***N***^_ denotes an ensemble average over a system containing ***n*** copies of each molecule.

While eq 31 is suitable for obtaining the chemical potential for small molecules where *N*_*m*_ ∼ 1, it is typically not suitable for computing the chemical potential of polymers where *N*_*m*_ is much larger. Calculating the chemical potential of polymers is difficult because the average in eq 31 is dominated by rare polymer configurations that correspond to small Δ*U*. For neutral polymers, these difficulties can be mitigated with the configurational bias Monte Carlo method^37−39^ or the chain increment method.^40^ Unfortunately, these methods struggle when the polymers are charged and the gradual insertion chains can result in systems with a nonzero net charge. The calculation of the chemical potential of polymers is further complicated when performing simulations on GPUs where many fast unbiased insertions can result in better performance than a small number of biased insertions that are performed more slowly.^41^ As a consequence, the calculation of the chemical potential in polymers is subject to numerous trade-offs, and no single method is expected to be optimal for the wide range of systems and conditions that we consider in this work.

In light of these considerations, we calculate the chemical potential using a method briefly described in section 11.2.2 of ref (5), where the excess chemical potential is given by
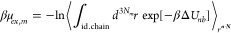
32where the integral includes the ideal chain configurations of a polymer with *N*_*m*_ beads, Δ*U*_nb_ is the change in nonbonded potential energy due to the insertion of a chain of molecule *m*, and ⟨··· ⟩_*r*^***n***·***N***^_ denotes an ensemble average over a system with ***n*** molecules. In this method, polymers with ideal chain statistics are randomly inserted into the system, and the change in nonbonded potential energy Δ*U*_nb_ arising from both intramolecular and intermolecular interactions involving this chain are computed. The primary advantage of eq 32 over eq 31 is that sampling is focused on ideal chain configurations instead of completely random configurations, many of which will have bonded potential energies Δ*U*_b_ that are large and have a negligible contribution to the averages in eq 31. Though eq 32 can be used for nonideal chains, the use of ideal chain insertions means that its sampling efficiency will improve as the real chains within the system become increasingly ideal. Since our model consists of soft interactions, we anticipate that chain configurations will be relatively ideal, and so eq 32 should provide an efficient route to calculate the chemical potential for the systems we consider in this work.

Our choice to calculate the chemical potential using eq 32 was also motivated by its ease of implementation, its applicability to both neutral and charged polymers, and its ability to be computed efficiently using GPUs. Moreover, we find that this method is reasonably efficient at estimating the chemical potential for the diverse range of polymeric systems that we consider in this work. Nonetheless, it is likely that eq 32 is not optimal for all systems and conditions that we examine, and so our results should be considered as such.

In field-theoretic simulations, the calculation of the chemical potential is more straightforward.^42^ In a field theoretic-simulation, *n*_*m*_ appears explicitly as a parameter in eq 10 and so the requisite *n*_*m*_ derivative of eq 9 can be explicitly evaluated
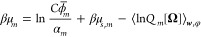
33where the first term is the ideal gas chemical potential, the second term is the self-contribution to the chemical potential that arises due to the *n*_*m*_ dependence of eq 12 converted to scaled units
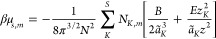
34and *Q*_*m*_[**Ω**] was defined in eq 17. An astonishing feature of field-theoretic simulations is that since *Q*_*m*_ is already calculated, the chemical potential is available without incurring any additional computational expense. This is in stark contrast to the Widom insertion-based methods required by particle simulations described above.

Despite the very different forms of eqs 32 and 33, these two expressions are formally equivalent and should yield identical values for the chemical potential of a system. We also note that knowledge of both the pressure and the chemical potential can be used to compute the Helmholtz free energy via . Since the chemical potential is available as a simple ensemble average in a field-theoretic simulation, this expression can be used to compute the free energy directly.^42^

### Performance Comparison of Particle and Field-Theoretic Simulations

Now that we have derived expressions for the pressure and chemical potential that can be used in both particle and field-theoretic simulations, we next sought to quantify the performance of each method. Specifically, we are interested in the computational effort required for each method to adequately sample the particle/field configurations so that the averages in eqs 24, 25, 32, and 33 converge to their equilibrium values.

In our analysis, we focus on two aspects of equilibration in a simulation. In the first, we focus on a simulation’s warm-up time, the time required for a simulation to begin sampling particle/field configurations with the proper equilibrium distribution. We quantify the warm-up time using block averages of the pressure and locate when these averages have reached their equilibrium value to within a relative error of 0.5%. The warm-up time is sensitive to the initial conditions used in the simulation, and so for simplicity we initialize our simulations randomly. In particle-based simulations, polymers are initialized as ideal chains with a randomly chosen center of mass. In field-theoretic simulations, all fields are initialized randomly from a Gaussian distribution with a mean of zero and a standard deviation of unity.

The second aspect of equilibration we focus on is the sampling time, the time required to sufficiently sample different particle/field configurations so that the averages ⟨··· ⟩_*r*^***n***·***N***^_ and ⟨··· ⟩_***w***,φ_ converge to their equilibrium values. We quantify the sampling time by computing a cumulative average of the chemical potential and then locating when this average is within a relative error of 0.5% of its equilibrium value. Clearly, these cumulative averages should only begin once a simulation is at equilibrium, and so the sampling time must always exceed the warm-up time. When performing these averages in particle-based simulations, we note that the ensemble average ⟨··· ⟩_*r*^***n***·***N***^_ for the chemical potential occurs within the logarithm in eq 32, and care should be taken that these averages are computed appropriately. For compactness, we will refer to both the warm-up time and the sampling time collectively as the equilibration time.

The equilibration time in FTS and MD will be quantified using wall time, the actual time required to run each simulation for equilibration to be reached. This wall time can be decomposed into two quantities: the number of steps to equilibrium and the wall time per step. On one hand, the number of steps to equilibrium is a *physical* quantity that depends on the underlying free energy surface , the initial condition used for the simulation, and the sampling efficiency of the underlying system dynamics. As a consequence, it is difficult it anticipate how the number of steps to equilibrium will scale with different model parameters. On the other hand, the wall time per step is a *computational* quantity that depends on the number of required floating point operations per step, which for both FTS and MD can be anticipated. In MD, this computational cost is dominated by the calculation of pairwise interactions in eq 6, which scales like  for uncharged particles and like  for charged particles if a cell-based neighbor list and PPPM electrostatics are used, respectively. In FTS, the computational cost is dominated by the solution of the propagators in eqs 18–20, which for pseudospectral methods scales like  where *N*_*t*_ = ∑_*m*_*N*_*m*_. The extent to which the equilibrium time follows these computational scalings provides an indication of whether this time is driven by computational or physical considerations within a simulation.

In our particle-based simulations, the pressure is computed every 10^3^ timesteps using eq 24, and the chemical potential is computed every 10^4^ timesteps using eq 32 with 10^3^ sample chain insertions. In order to efficiently perform these chain insertions on the GPU, many chain configurations were transferred to the GPU simultaneously, and their potential energies were computed in parallel. We also precomputed a large library of chain configurations prior to the start of each simulation to avoid needing to compute these configurations on-the-fly during a simulation. In our field-theoretic simulations, both the pressure and chemical potential are computed every 10^2^ timesteps using eq 25 and eq 33, respectively. All calculations are performed for five independent replicates that are initialized from different random initial conditions and are evolved using different random seeds. Performance comparisons of particle-based and field-theoretic simulations are performed on identical hardware consisting of dual Intel 8268 CPUs and a single Nvidia V100 GPU. Particle-based simulations are performed using HOOMD-blue v3.11,^43^ and field-theoretic simulations are performed using OpenFTS, an in-house code developed by our group.

## Results

Now that we have formulated a general model that can be examined in both particle and field-theoretic simulations, we next turn to our central objective where we compare the predictions and performance of the two methods. In the sections that follow, we consider four different polymeric systems: (1) a homopolymer solution or melt, (2) a diblock copolymer melt, (3) polyampholytes in implicit solvent, and (4) polyelectrolyte gels with explicit solvent and salt ions. For each of these systems, we first demonstrate how our general model simplifies in each of these limiting cases and how our model relates to past field-theoretic models of these systems. We next compare the predictions of particle and field-theoretic simulations for each system and identify when the two methods are in quantitative agreement. When performing these comparisons, we choose to specify model parameters in the reduced units that appear in the field-theoretic representation in eq 10 (e.g., *B*, *C*, and *E*) instead of the units that appear in the particle-based representation in eq 3 (e.g., *u*_0_, ρ_0_, and *l*_*b*_). As we demonstrate shortly, the use of reduced units will allow us to collapse our results onto universal curves that are independent of *N*. Finally, we will compare the computational expense of the two methods in order to understand when the performance of each method is superior.

### Homopolymer Melt or Solution

The first system that we consider is a homopolymer melt or solution. This system consists of a single molecule type (*M* = 1) consisting of a single species (*S* = 1) polymerized into a chain of length *N*_1_ = *N*. Since there is only one molecule and species type, we let *n*_1_ = *n*, *a*_1_ = *a*, *b*_1_ = *b*, λ_1_ = 1, and *z*_1_ = 0 so that the species is uncharged. For this system, the field theory given in eqs 9 and 10 simplifies considerably with ***w*** = {*w*_1_}, ***X*** = ((*BC*)^1–1/ν^), trivial eigenvector ***A*** = (1), eigenvalue Λ_1_ = (*BC*)^1–1/ν^, γ_1_ = *i*, and rank *R* = 1. Moreover, since there are no charged interactions, the φ field does not appear in the theory. These simplifications yield the following field theory:
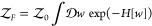
35
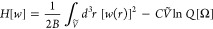
36where Ω = *i*Γ★*w*(*r*)/*N* and *n* = *CṼ*. We note that eqs 35 and 36 are consistent with past work on this homopolymer model.^30,44^

**Figure 1:**
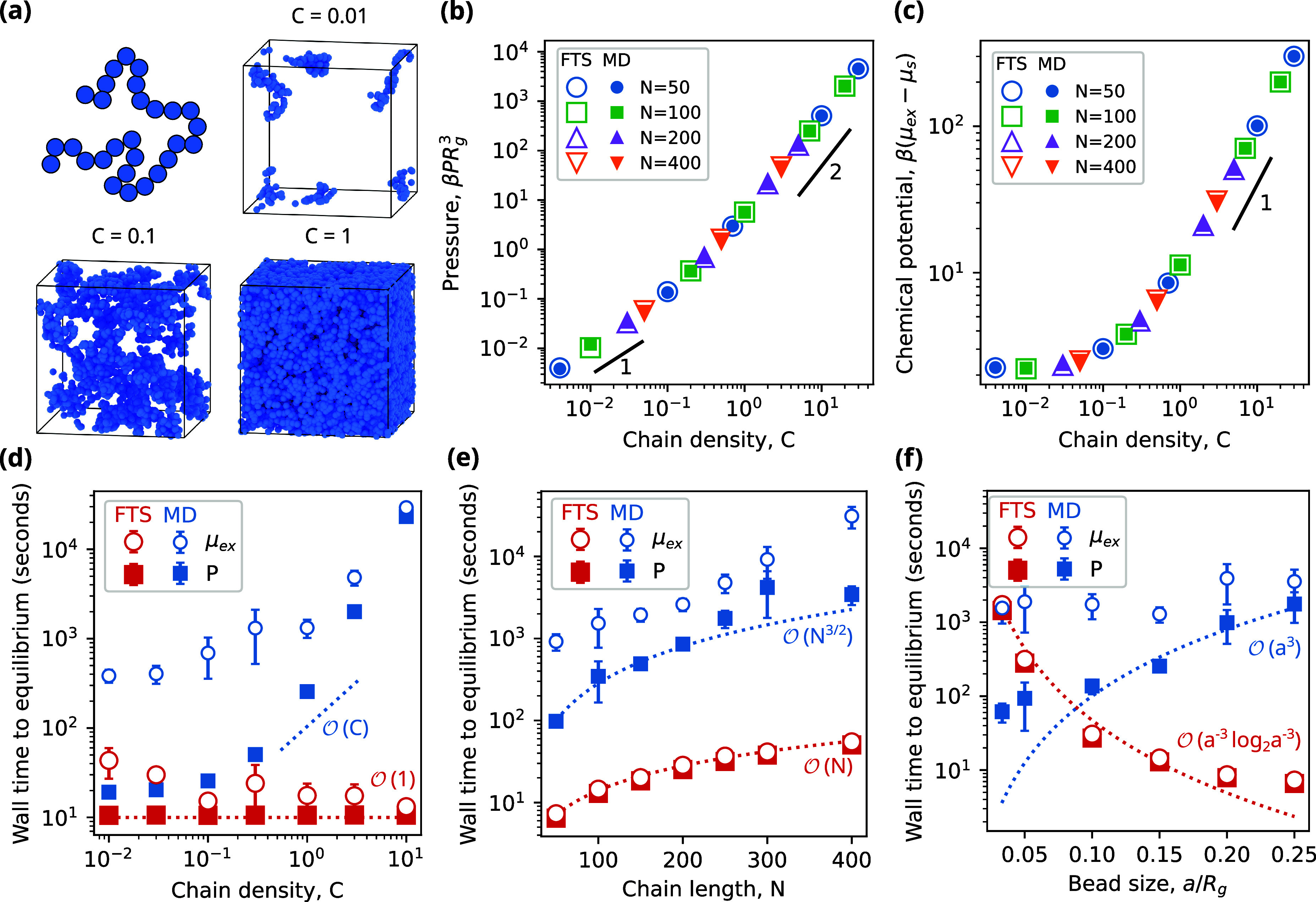
Comparison of FTS and MD for a homopolymer melt and solution. (a) Simulation snapshots of homopolymer model for different chain densities, *C*. FTS and MD give equivalent results for the (b) pressure and (c) chemical potential across different densities and chain lengths, *N*. Performance comparison of FTS and MD for different (d) densities, (e) chain lengths, and (f) characteristic bead sizes. Dotted lines show scaling of computational cost per time step.

In our analysis of this model, we assume cubic boxes with *L*_*x*_ = *L*_*y*_ = *L*_*z*_ = 6.4*R*_g_, *B* = 10, *a* = 0.15*R*_g_ and spatial discretization *M*_*t*_ = 48^3^. This leaves two unspecified parameters, the chain density *C* and chain length *N*. At high values of *C* this model corresponds to a polymer melt, while at low values of *C* this model corresponds to a polymer solution with the solvent represented implicitly (Figure 1a). We performed a series of FTS and MD simulations across a range of chain densities and chain lengths and measured the pressure (Figure 1b) and chemical potential (Figure 1c). For all conditions considered, the results from FTS and MD are in excellent agreement.

One noteworthy feature of these results is the agreement of the chemical potential in the regime where the density is high (e.g., *C* ≥ 1) and the polymers are long (e.g., *N* ≥ 200). Though this regime is where Widom insertion schemes typically fail, we observe that our insertion scheme described in eq 32 nonetheless works quite well and yields exact agreement with FTS. This agreement suggests that our relatively simple insertion scheme is sufficiently accurate for this system and that more elaborate methods^37−40^ are not required.

We next consider the computational performance of FTS and MD. We first consider how the wall time to equilibrium scales with respect to *C* for *N* = 100 (Figure 1d). In our analysis, we quantify two aspects of equilibration in a simulation: the warm-up time using the pressure and the sampling time using the chemical potential (see Methods). When considering both the pressure and chemical potential, the performances of FTS and MD are comparable when *C* ≤ 10^–1^ but differ significantly as *C* is increased. Whereas the equilibration time in FTS follows approximate  scaling, the equilibration time in MD increases sharply as the density is increased with a scaling slightly larger than . For large densities, the performance difference between FTS and MD can exceed 3 orders of magnitude.

We also examine the equilibration time of FTS and MD with respect to chain length for *C* = 1 (Figure 1e). Both FTS and MD generally follow the expected computational scalings of  and , respectively. We find that FTS is more efficient than MD for all chain lengths examined from *N* = 50 to *N* = 400. Finally, we examine the performance of FTS and MD with respect to *a*, the characteristic size of beads in the system, for *C* = 1 and *N* = 100 (Figure 1f). Larger values of *a* increase the cost of MD due to the larger required pair cutoff *r*_*c*_ yet decrease the cost of FTS due to the lower number of required grid points *M*_*t*_. For example, as *a* is increased from 0.025 to 0.25, the pair cutoff in MD increases from *r*_*c*_ = 0.65*b* to *r*_*c*_ = 6.5*b*, while the spatial discretization in FTS decreases from *M*_*t*_ = 256^3^ to *M*_*t*_ = 27^3^. When equilibrating the pressure, we find that MD is more efficient for small bead sizes *a* ≤ 0.05*R*_g_, while FTS outperforms MD for bead sizes that are larger. When calculating the chemical potential, we find that FTS is equal to or faster than MD for all of the *a* examined.

### Diblock copolymer melt

The next system that we consider is a melt of linear diblock copolymers. This system consists of *n*_1_ = *n* copies of a single polymer type (*M* = 1) with length *N*_1_ = *N*. This polymer consists of *f N* beads of species 1 followed by (1 – *f*)*N* beads of species 2, where *f* is the block fraction of species 1. For simplicity, we let these two species have equal statistical segment lengths *b*_1_ = *b*_2_ = *b*, characteristic sizes *a*_1_ = *a*_2_ = *a*, and zero net charge *z*_1_ = *z*_2_ = 0. The incompatibility between the two species is given by χ_12_ = χ. For this two-component system,
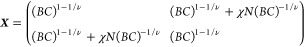
37which for nonzero χ has rank *R* = 2, eigenvectors
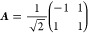
38and eigenvalues Λ_1_ = −*χN*(*BC*)^−1/ν^ and Λ_2_ = 2(*BC*)^1–1/ν^ + *χN*(*BC*)^−1/ν^. For these eigenvalues, γ_1_ = 1 and γ_2_ = *i*. After applying these simplifications, the field theory given in eqs 9 and 10 becomes
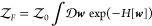
39
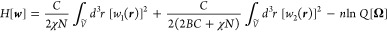
40where ***w*** = {*w*_1_, *w*_2_} and . This diblock model is similar to prior results^21,44^ except that the first two terms in eq 40 contain an extra factor of 1/2, which can be absorbed into the fields *w*_1_ and *w*_2_ without consequence. For this rescaling, *w*_1_ becomes equivalent to the exchange field typically denoted by *w*_–_ and *w*_2_ becomes the pressure-like field typically denoted by *w*_+_. In the results that follow, we let *B* = 20, *C* = 5, *a* = 0.15*R*_g_, λ_1_ = 1, and λ_2_ = 0.5.

To demonstrate the equivalence of FTS and MD for this system, we consider a symmetric diblock copolymer (i.e., *f* = 0.5) as χ is varied above and below its order–disorder transition (ODT), which for our model parameters is located at χ_*ODT*_*N* = 11.4.^44^ We use cubic boxes with *L* = 6.4*R*_g_ for χ*N* < χ_ODT_*N* and *L* = 2L* for χ*N* ≥ χ_ODT_*N*, where L* is the domain spacing calculated for this model using SCFT. Though the domain spacing differs slightly between SCFT and FTS due to fluctuations,^42^ these effects are small for the conditions we consider here.^21^ We also found it necessary to increase the pair cutoff in MD from our typical value of *r*_*c*_ = 6.4*a* to a higher value of *r*_*c*_ = 7.5*a*.

**Figure 2:**
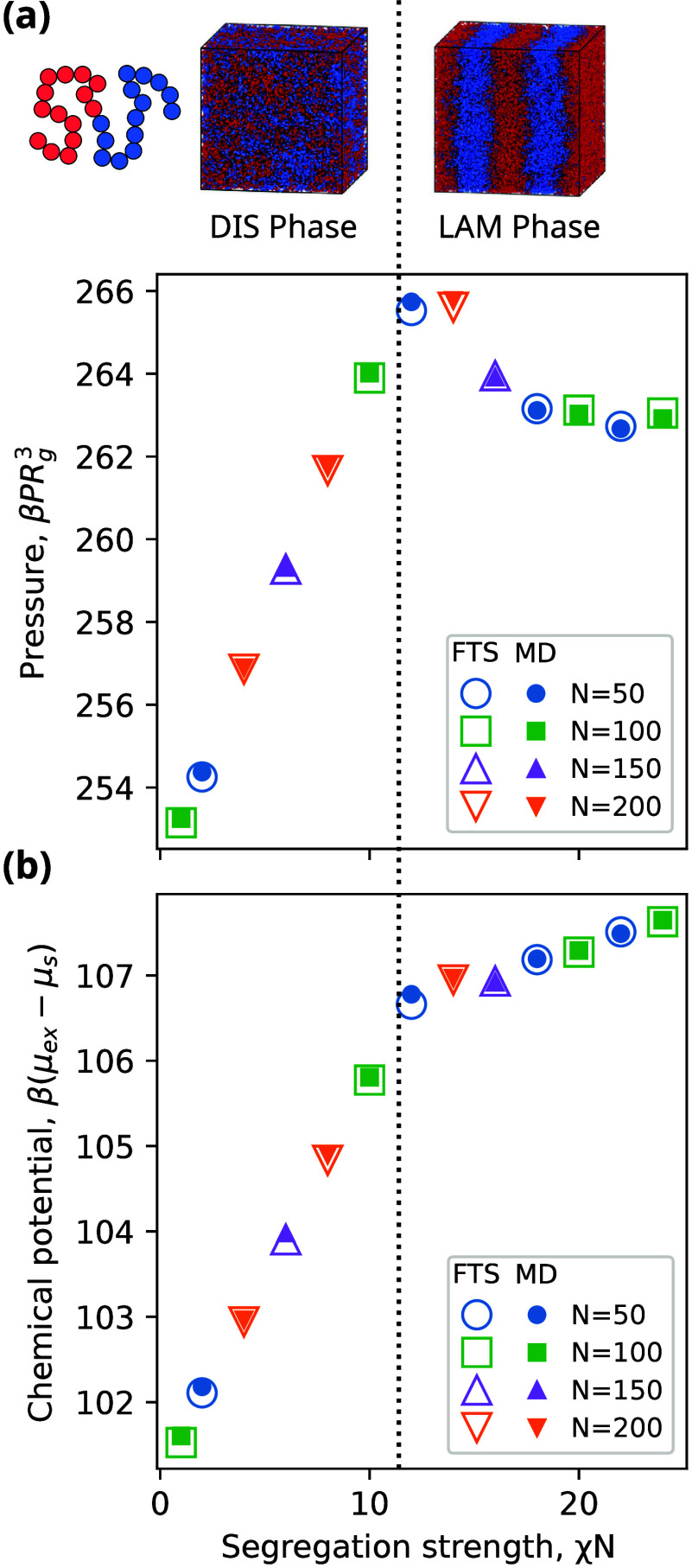
Equivalence of FTS and MD for a symmetric diblock copolymer melt. Predictions of the (a) pressure and (b) chemical potential are identical between the two methods for all segregation strengths (*χN*) and chain lengths (*N*) examined.

We calculate the pressure and chemical potential in both FTS and MD and observe that they agree well across a range of *χN* and *N* values (Figure 2). The excellent agreement between the chemical potential is especially noteworthy since the system conditions (i.e., long chains, high densities, and microphase separation) are typically challenging for Widom insertion schemes. As with Figure 1c, this result suggests that our relatively simple insertion scheme in eq 32 is sufficient to accurately calculate the chemical potential in MD simulations.

**Figure 3:**
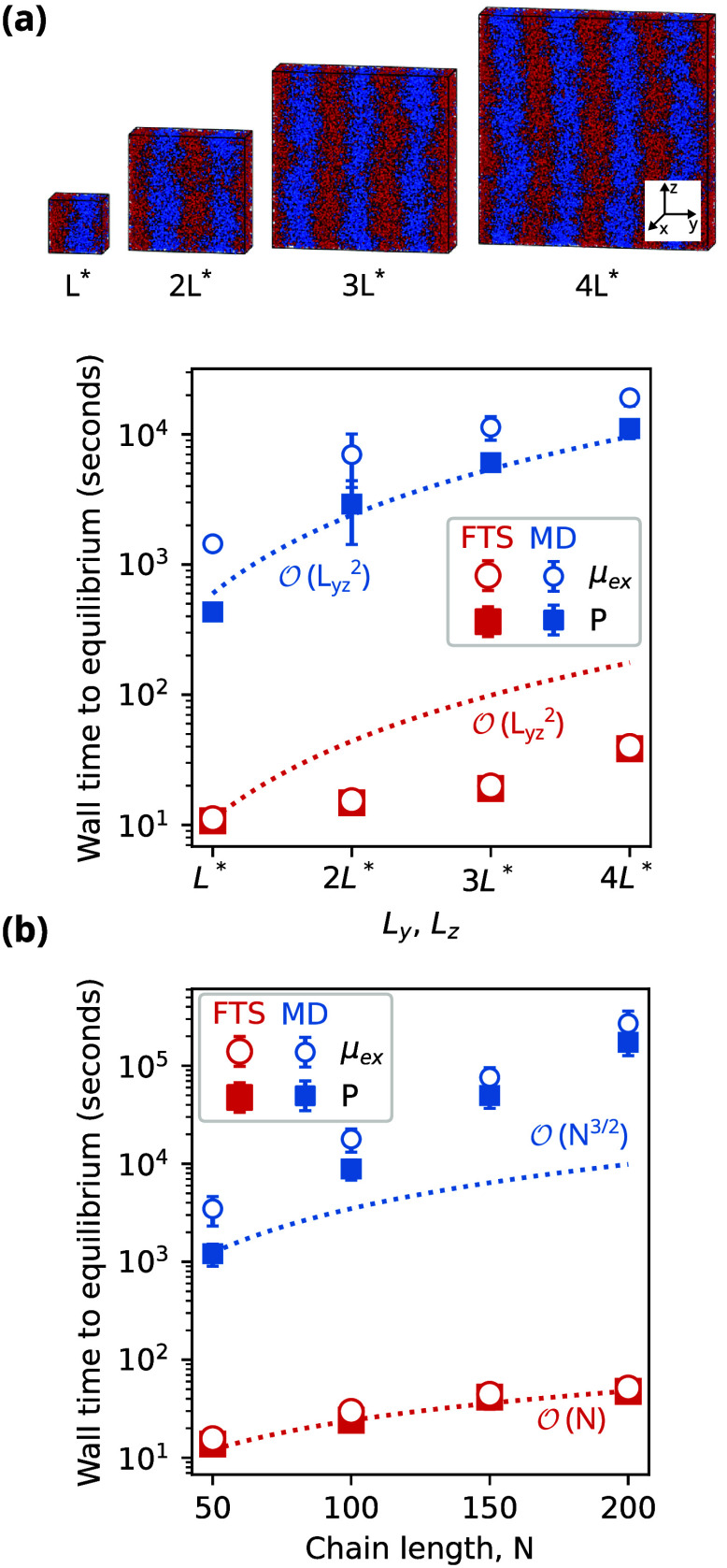
Performance comparison of FTS and MD for a symmetric diblock copolymer melt. Effect of (a) system size and (b) chain length on the wall time to equilibrium for pressure and chemical potential. Dotted lines show scaling of computational cost per time step.

We also quantify the computational performance of FTS and MD for diblock copolymer melts as a function of both the system size and chain length (Figure 3). To examine the effect of system size, we use a noncubic box with *L*_*x*_ = 3*R*_g_ and *L*_*y*_ = *L*_*z*_ = jL*, where *j* is a positive integer. This box was chosen to avoid the formation of defects and long-lived metastable states in the *x* dimension, which could lead to large variation between different replicates. For these calculations, *N* = 50 and *χN* = 16.

For all system sizes examined, FTS reaches equilibrium faster than MD (Figure 3a). These results are consistent with our earlier results in Figure 1d, where we observed that FTS is favored over MD at the large densities that correspond to the melt conditions considered here. An interesting feature of our results is that the equilibration time of FTS is faster than the computational scaling of . This indicates that even though the computational expense per step is increasing with *L*_*xy*_^2^, the number of steps to equilibrium is decreasing more rapidly, which results in a performance scaling that only increases slightly with *L*_*xy*_. This result contrasts the performance of MD which follows the computational scaling of .

Our calculations also demonstrate that FTS reaches equilibrium faster than MD for all chain lengths from *N* = 50 to 200 for *χN* = 16 and *L* = L* (Figure 3b). In FTS, the wall time to equilibrium scales with the computational scaling of . In MD, the wall time to equilibrium increases more rapidly than the expected computational scaling of  and indicates that more steps are needed to reach equilibrium as the length of the polymer chains increase.

### Polyampholyte in Implicit Solvent

We next consider polymeric systems that include long-range Coulombic interactions. In particle-based simulations, Coulombic interactions must be handled using specialized Ewald-based techniques that considerably increase the computational expense and complexity of these simulations.^4,5^ In contrast, Coulombic interactions in field-theoretic simulations appear as the semilocal square gradient term |∇φ|^2^ in eq 10, which can be evaluated locally using pseudospectral methods.^8^ As a consequence, field-theoretic simulations with Coulombic interactions are only marginally more expensive than simulations where only short-range interactions are present. This advantageous feature of field-theoretic simulations suggests that they might be favored over particle simulations when long-range Coulombic interactions are present.

The first charged polymeric system that we consider is a diblock polyampholyte in implicit solvent. This system consists of *n*_1_ = *n* copies of a single molecule type (*M* = 1, *S* = 2) with length *N*_1_ = *N* consisting of *fN* beads of species 1 followed by (1 – *f*)*N* beads of species 2, where *f* is the fraction of beads that are of species type 1. Each species type has equal and opposite charge (*z*_1_ = +*z*, *z*_2_ = −*z*), equal characteristic size *a*_1_ = *a*_2_ = *a* and equal statistical segment length *b*_1_ = *b*_2_ = *b*. We let χ_12_ be 0 so that short-range interactions between different bead types are equal.

For this system, ***X*** = ((*BC*)^1–1/ν^)**1**, which has rank *R* = 1, nonzero eigenvalue Λ_1_ = (*BC*)^1–1/ν^, eigenvector
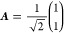
41and γ_1_ = *i*. The presence of charged species in this system leads to a nonzero charge density and the appearance of the φ field in the theory. With these considerations, eqs 9 and 10 simplify to
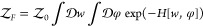
42
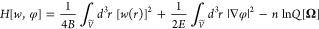
43where ***w*** = {*w*} and the fields **Ω** = {Ω_1_, Ω_2_} are given by  for *K* = 1, 2. As we observed for the diblock in eq 40, this model is the same as past results^7,9,32,33^ if a factor of  is absorbed into the *w* field.

Now that a suitable polyampholyte model has been obtained, we examine the equivalence of FTS and MD. For our analysis, we consider symmetric polyampholytes with *f* = 0.5, excluded volume strength *B* = 1, electrostatic strength *E* = 1000, cubic boxes with *L* = 9.6*R*_g_, characteristic bead size *a* = 0.15*R*_g_, mobility coefficients λ_1_ = 1, λ_φ_ = 1, and spatial discretization *M*_*t*_ = 64^3^.

**Figure 4:**
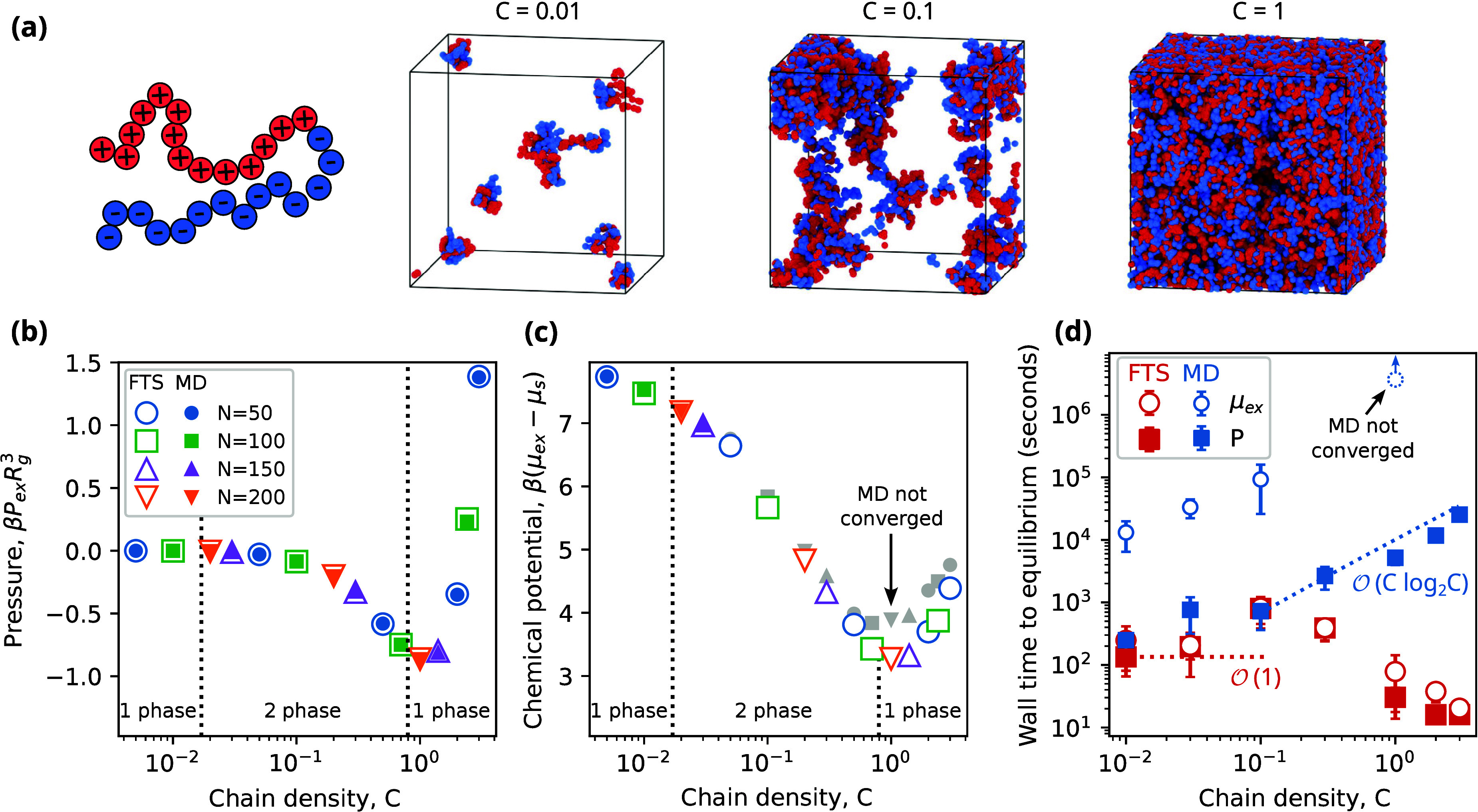
Comparison of FTS and MD for a polyampholyte solution. (a) Simulation snapshots of the model for different chain densities, *C*. (b) FTS and MD yield equivalent values of the pressure across all densities and chain lengths, *N*. (c) Chemical potentials from FTS and MD agree well at low density but differ as density increases. These differences are attributed to sampling errors in MD when the density is large. (d) Performance comparison of FTS and MD for different densities. Dotted lines show scaling of computational cost per time step.

A remaining parameter is the chain density *C*, which controls whether the system will exist as a dilute, phase-separated, or dense phase (Figure 4a). We compute the pressure for both FTS and MD across a range of *C* and chain lengths *N* and observe the two methods to be in excellent agreement across the entire range of parameters (Figure 4b).

When comparing the chemical potential, we find that FTS and MD agree well at low chain densities *C* ≤ 0.04 (Figure 4c). As the chain density increases, the chemical potential estimated by MD is systematically higher than that obtained by FTS. Despite our best efforts, including over 3 × 10^6^ seconds of simulation time, we were unable to converge these MD calculations to agree with FTS. We attribute this systematic difference due to sampling errors in MD and the challenges of locating chain configurations with small Δ*U*_nb_ that dominate the average in eq 32.

We next compared the equilibration time between FTS and MD for this polyampholyte system (Figure 4d). When considering the pressure, the equilibration times of FTS and MD are comparable at low *C* but differ significantly as *C* increases. Equilibration in MD becomes slower with increasing *C* and generally follows the computational scaling of . In contrast, equilibration in FTS becomes faster with increasing *C* and is even better than the computational scaling of . For large *C* the performance difference between FTS and MD is approximately 3 orders of magnitude.

When considering the chemical potential, the performance differences between FTS and MD become even more stark. In FTS, the equilibration time of the chemical potential is comparable to that of the pressure and leads to a marginal increase in the expense of a simulation. In MD, calculation of the chemical potential considerably increases the cost of a simulation, typically by several orders of magnitude. This cost, combined with MD’s computational scaling of , makes it intractable for us to accurately calculate the chemical potential in dense systems of charged polymers. Based on our calculations, the performance difference between FTS and MD in this regime exceeds 4 orders of magnitude. While it is possible that more sophisticated chemical potential methods^37−40^ might narrow this performance gap between FTS and MD, we find it unlikely that any particle-based method will be competitive with FTS for dense polymeric systems with Coulombic interactions.

### Polyelectrolyte Gel with Explicit Solvent and Salt Ions

The final system we consider is a polyelectrolyte gel containing explicit solvent (i.e., water) and salt ions. This system consists of a polymer solution with two symmetric triblock copolymers that carry oppositely charged end-blocks and hydrophilic midblocks.^45−47^ The electrostatic complexation of end blocks drives the formation of microphase-separated domains, which serve as physical cross-links within these materials. This class of materials has received considerable attention over the past decade, which has been summarized in a recent review.^48^

In order to represent this system in our generalized polymer model, six different species types are required (*S* = 6). Species 1 and 2 correspond to the positive and negatively charged end-blocks of the triblock copolymers that carry charges *z*_1_ = +*z* and *z*_2_ = −*z*, respectively. Species 3 corresponds to the neutral hydrophilic midblock with *z*_3_ = 0, while species 4 and 5 correspond to free cations and anions and carry charges *z*_4_ = +*z* and *z*_5_ = −*z*. Species 6 corresponds to the solvent that is uncharged *z*_6_ = 0. For simplicity, the characteristic sizes and statistical segment length of all species are assumed to be equal, *a*_*i*_ = *a*, *b*_*i*_ = *b* for *i* ∈ {1, ···, 6}.

In addition to long-range Coulombic interactions, these species also interact via short-range interactions. For our calculations, we let the end-blocks have a slight incompatibility with the midblocks χ_13_ = χ_23_ = 0.03 and let the polymer species have a small incompatibility with the solvent χ_16_ = χ_26_ = χ_36_ = 0.06. All other χ_*ij*_ values between different pairs of species are set to zero. For these short-range interactions, the resulting 6 × 6 ***X*** matrix has rank *R* = 4 and so four fields ***w*** = {*w*_1_, *w*_2_, *w*_3_, *w*_4_} are required to decouple the short-range interactions. A fifth φ field is required to decouple the electrostatic interactions.

To fully specify this system, we combined these six species together to form five molecules (M = 5). Molecules 1 and 2 are both triblock copolymers with equal length *N*_1_ = *N*_2_ = 2*N* consisting of end-blocks of species 1 or 2 of length *fN* separated by a midblock of species 3 of length 2*N*(1-*f*). Molecules 3, 4, and 5 correspond to free cations, anions, and solvent, respectively, each of which consist of a single bead *N*_3_ = *N*_4_ = *N*_5_ = 1. We constrain the cation and anion volume fractions to be equal to ϕ̅_3_ = ϕ̅_4_ and set the total salt volume fraction ϕ_*s*_ = ϕ̅_3_ + ϕ̅_4_ = 0.02. We also constrain the triblock copolymers to have equal volume fraction ϕ̅_1_ = ϕ̅_2_ so that the overall system has zero net charge and specify the total polymer volume fraction as ϕ_*P*_ = ϕ̅_1_ + ϕ̅_2_. For the calculations that follow, we let *f* = 0.2, *N* = 50, *a* = 0.15 *R*_g_, *B* = 1, and *C* = 5 and use cubic boxes with *L* = 9.6*R*_g_ with spatial discretization *M*_*t*_ = 64^3^. We also use mobility coefficients λ_1_ = 0.3, λ_2_ = 0.2, λ_3_ = 0.2, λ_4_ = 1, and λ_φ_ = 1.

The only remaining parameters needed to specify this model are the polymer volume fraction, ϕ_P_, and the reduced Bjerrum length, *E*. While our model permits ϕ_P_ and *E* to be chosen independently, in any physical system, these parameters will be coupled due to the unequal dielectric constant of the polymer and solvent. An elegant solution to this problem is to instead allow the solvent to carry a freely rotating permanent dipole and to use the vacuum Bjerrum length in the definition of *E* so that the effective dielectric constant emerges from the explicit dielectric screening of the solvent.^8,49−51^ Unfortunately, such a solution is beyond the scope of this work, and so for simplicity we assume that ϕ_P_ and *E* are not coupled and can be specified independently.

**Figure 5:**
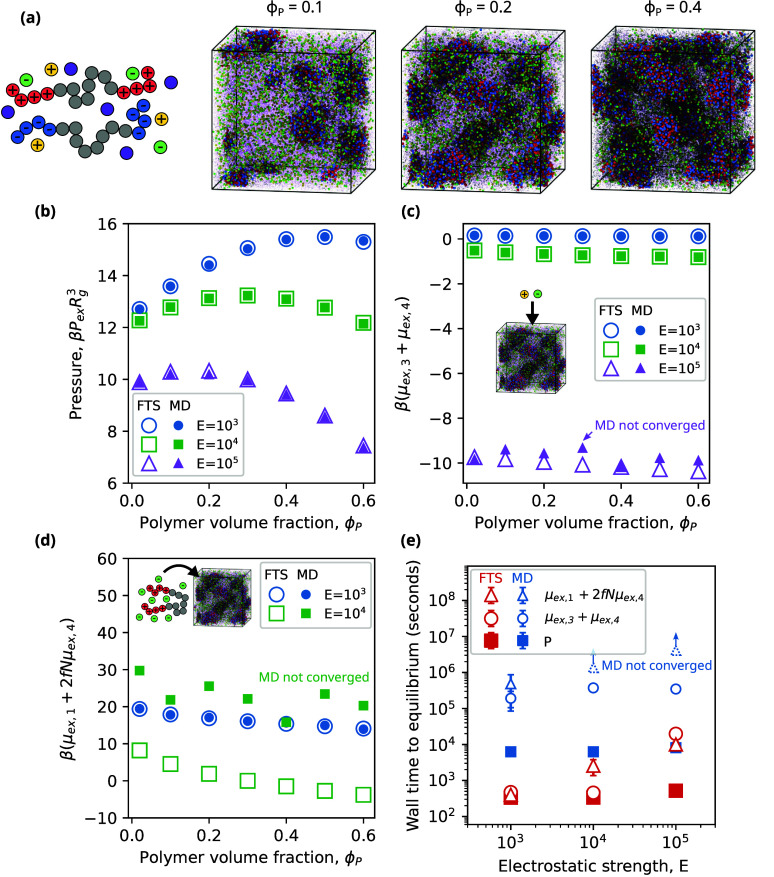
Comparison of FTS and MD for a polyelectrolyte gel with explicit solvent and salt ions. (a) Simulation snapshots for different polymer volume fractions, ϕ_*P*_. (b) FTS and MD give equivalent results for the pressure across all polymer volume fractions and electrostatic strengths, *E*. (c) Chemical potentials of salt ions are equivalent between FTS and MD for *E* ≤ 10^4^. MD struggles to converge for *E* = 10^5^ and deviates from FTS predictions. (d) Chemical potentials for polycations and associated counterions agree well between FTS and MD for *E* = 10^3^. For higher *E*, MD values do not converge due to sampling errors. (e) Performance comparison of FTS and MD for different electrostatic strengths.

In order to qualitatively characterize this system, we begin by varying ϕ_P_ for *E* = 10^5^ (Figure 5a). This value of *E* is sufficiently high so that the end groups of the triblock copolymers associate to form complex coacervate core micelles. As ϕ_P_ increases, the morphology of these micelles first transitions to spheres and then to cylinders. For the sake of brevity, we do not characterize the precise values of ϕ_P_ that correspond to these transitions. We next examine the equivalence of FTS and MD by computing the pressure across a range of ϕ_P_ and electrostatic strengths *E* (Figure 5b). The agreement between the FTS and MD across this range of parameters is excellent.

The comparison of the chemical potential was slightly more complicated. Since both the polymers and salt ions carry a net charge, we are unable to calculate the chemical potential of these molecules independently in MD using eq 32 since the inserted molecules will lead to a system with an overall net charge. This issue is easily addressed by performing insertions of pairs or groups of molecules so that the overall insertion carries a zero net charge. For example, an insertion might consist of a polycation and its accompanying 2*fN* negative counterions. A slight disadvantage of this approach is that it yields the overall chemical potential due to the charge-neutral insertion and not that of the individual molecules. For instance, the insertion of a polycation and its counterions yields μ_*ex*,1_ + 2*fNμ*_*ex*,4_ via eq 32 and not μ_*ex*,1_ or μ_*ex*,4_ directly. In contrast, the calculation of the chemical potential in FTS has no such limitation, and eq 33 can be used to calculate the chemical potential for each molecule in the system without issue.

With these considerations, we first compare the chemical potentials in FTS and MD for the positive and negative salt ions μ_*ex*,3_ + μ_*ex*,4_ (Figure 5c). For lower values of electrostatic strength (*E* = 10^3^ to 10^4^), the agreement between FTS and MD is excellent across the range of polymer volume fractions examined. FTS and MD are also generally in agreement for a higher electrostatic strength (*E* = 10^5^), but the results from MD are substantially noisier. We attribute this noise to sampling errors in MD and the challenges in adequately estimating the ensemble average in eq 32 as *E* increases.

We also compare the chemical potential in FTS and MD for the polycation and its accompanying counterions μ_*ex*,1_ + 2*fNμ*_*ex*,4_ (Figure 5d). While FTS and MD agree well for *E* = 10^3^, we see a systematic deviation for *E* ≥ 10^4^ even for long simulations times in excess of 10^6^ seconds. These errors are so severe for *E* = 10^5^ that these data have been omitted for clarity. As before, we attribute these differences to sampling errors in MD and the challenges of sampling configurations with small Δ*U*_nb_ values that dominate the average in eq 32. These errors are particularly severe for this system because both polymer and counterion configurations must be adequately sampled. While these challenges can be overcome for *E* = 10^3^, we are unable to achieve agreement between FTS and MD for *E* ≥ 10^4^.

Finally, we examine the equilibration time between FTS and MD for this system with ϕ_*P*_ = 0.4 and *E* = 10^3^ – 10^5^ (Figure 5e). When equilibrating the pressure, FTS reaches equilibrium approximately an order of magnitude faster than MD across all *E* examined. We also observe that the equilibration time of the pressure increases slightly as *E* increases. When equilibrating the chemical potential, FTS is considerably faster than MD and exceeds 3 orders of magnitude for all *E* examined. In FTS, the equilibration time for the chemical potential of pairs of salt ions (μ_*ex*,3_ + μ_*ex*,4_) is comparable to that of the polycation and its counterions (μ_*ex*,1_ + 2*fNμ*_*ex*,4_) and increases slightly with increasing *E*. In contrast, the equilibration times in MD for these two chemical potentials are very different. We found that the computational demands required to estimate the chemical potential of the polycation and counterions (μ_*ex*,1_ + 2*fNμ*_*ex*,4_) were considerably higher than those required to estimate that of the salt ion pairs (μ_*ex*,3_ + μ_*ex*,4_). While μ_*ex*,3_ + μ_*ex*,4_ would eventually converge in days of wall time, we found that μ_*ex*,1_ + 2*fNμ*_*ex*,4_ would not converge even for wall times exceeding months. As with our discussion of Figure 4d, part of the poor performance of MD likely results from sampling inefficiencies in eq 32 which might be mitigated with alternative methods.^37−40^ Nonetheless, since the time required to estimate the chemical potential in MD is many orders of magnitude larger than in FTS, we are not optimistic that these alternative methods will substantially narrow the performance gap that exists between FTS and MD.

## Discussion and Conclusion

In this work, we present a systematic and comprehensive comparison of molecular dynamics and field-theoretic simulations. To perform this comparison, we have first derived a generalized model that can be used to simulate a wide range of polymeric systems encompassing homopolymers, block copolymer melts, polyampholyte solutions, and polyelectrolyte gels. Using this generalized model, we then perform equivalent particle and field-theoretic simulations and compare the results and performance of the two methods.

Our first key finding is that particle and field-theoretic simulations give equivalent results across this broad range of systems. This result provides compelling evidence that field-theoretic simulations are thermodynamically equivalent to particle simulations and that these two methods are complementary tools for sampling the same underlying partition function. While particle-based simulations of polymers are ubiquitous across the literature, field-theoretic simulations are less widely used. As a consequence, field-theoretic simulations are often viewed with some degree of skepticism by researchers who are not familiar with the details of the method. Our results should provide some degree of comfort to these nonpractitioners that the theory and numerical methods that underpin field-theoretic simulations are fundamentally sound and that the method is truly equivalent to more widely used techniques like molecular dynamics.

The second key finding of our work is that field-theoretic simulations reach equilibrium equal to or faster than particle simulations for nearly all conditions studied. When the system density is low, the polymer chains are short and interactions are short-range, these advantages are small, and FTS and MD reach equilibrium in a comparable amount of time. However, when the system density becomes large, the chains become long, or the system becomes large, FTS can be orders of magnitude faster than MD, despite giving identical results. We also find that field-theoretic simulations are preferred for systems containing long-range Coulombic interactions, in part because the method does not require Ewald-based methods that are necessary to incorporate these interactions into particle simulations. FTS also provides facile access to the chemical potential and does not require the Widom-like insertions needed to calculate the chemical potential in MD. When all of these considerations are taken together, we find that FTS can efficiently equilibrate dense, charged polymeric systems that are essentially intractable with MD.

Part of the explanation for the superior performance of FTS derives from its favorable computational scaling. In FTS, since the computational cost per step scales like  with respect to density and  with respect to chain length, it is reasonable that FTS would be preferred over MD when the density is large or the chains are long. However, our results also indicate that computational scaling alone is insufficient to explain the superior performance of FTS. For example, our results for large (Figure 3a) or charged (Figure 4d) systems show that FTS reaches equilibrium faster than what would be expected based on computational scaling alone. These results suggest that the FTS method is able to locate and sample the equilibrium state in fewer steps, which also leads to an increase in performance. In contrast, MD tends to either follow its expected computational scaling (e.g., Figure 3a, Figure 4d) or to scale more poorly (e.g., Figure 1d and e, Figure 3b). Since the dynamics in MD correspond to physical processes within a system, it is natural to expect that more simulation steps might be required to locate and sample equilibrium as system conditions are changed. As a consequence, the computational scaling in MD can be considered a best-case scenario. Alternatively since the time in FTS is fictitious, the dynamics in these simulations are not restricted to physically meaningfully trajectories and so FTS can locate and sample equilibrium configurations using alternate pathways that are inaccessible to MD. We anticipate that it is the availability of these nonphysical relaxation pathways that is responsible for FTS’s superior approach to and sampling of equilibrium. When this improved sampling is combined with its favorable computational scaling, FTS can become orders of magnitude faster than equivalent MD simulations.

While our results demonstrate that FTS can be superior to MD across a wide range of systems and conditions, it is important to emphasize that there are also situations where FTS is expected to perform poorly relative to MD. In our work here, we have focused on parameters with soft excluded volume interactions where *βu*_*ij*_ ≪ 1 and substantial overlap between particles is permitted. We restricted our study to soft interactions because the numerical methods required to perform FTS are more mature in this regime and so no new numerical methods were required to perform the simulations throughout this work. Nonetheless, based on our experience, we have found that FTS can become considerably more challenging as the excluded volume interactions between particles increase. These challenges include both numerical difficulties associated with stabilizing the CL dynamics in eqs 22 and 23 and sampling difficulties associated with long-lived metastable basins where the imaginary parts of operators do not average to zero.^36^ Due to these difficulties, FTS is not well-suited to study problems where strong excluded volume interactions are important, such as packing or crystallization. In contrast, MD simulations are easily and routinely performed when strong excluded volume interactions are present and are expected to outperform FTS in this regime.

Another limitation of FTS is that it places somewhat onerous restrictions on the functional form of potential energy *U*(***r***^***n***·***N***^). These restrictions include the requirements that the potential energy must consist of a sum of pairwise interactions (c.f. eq 6) and that the nonbonded pair-potential *u*_nb_(*r*) be finite at *r* = 0, be positive-definite, and have a functional inverse.^7,8^ These requirements amount to the condition that the Fourier transform of *u*_nb_(*r*)
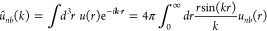
44exists and is positive for all *k* = |***k***|.^8^ Due to these restrictions, FTS is not currently possible with several pair-potentials that are ubiquitous in particle-based simulations, such as the Lennard-Jones and Weeks–Chandler–Andersen potentials.^52,53^

Though the inability to perform FTS with these common pair-potentials is a current limitation of the method, several strategies have recently been proposed to address these challenges. The essence of these strategies is to perform minor modifications to nonpositive-definite pair-potentials so as to render them positive-definite and thus amenable to FTS. If these modifications are sufficiently small, they can be performed with minimal effects on the thermodynamic and structural properties of the system. For example, the requirement of finite *u*_nb_(0) is easily addressed by regularizing the potential in the vicinity of *r* = 0 while leaving the remainder of the potential intact,^7,28,30^ as we have done with the Coulomb interactions in eq 3. Other strategies include approximating the pair-potential by an expansion in basis functions that are individually positive-definite and invertible (e.g., Gaussians)^8^ or to remap the desired potential to qualitatively similar potentials that are positive-definite like the Morse potential.^54−56^ Another strategy is to instead formulate FTS using a density-explicit field theory that does not require *u*_nb_(*r*) to be positive-definite and is not restricted to two-body interactions.^7,8^ Though these approaches are still in their infancy, our work here provides considerable motivation for additional efforts in these directions: if FTS can be achieved with more expressive pair-potentials, then these calculations could be orders of magnitude faster than equivalent particle-based simulations.

While our results provide the most comprehensive comparison of FTS and MD to date, there are nonetheless systems and regimes that were inevitably omitted from our study. For the sake of completeness, we briefly highlight several directions that we feel are of particular interest for future studies. First, we see great value in comparing the performance of FTS and MD on the basis of structural properties (e.g., the structure factor) instead of only thermodynamic properties (i.e., pressure, chemical potential) as we have done here. We anticipate that structural properties could provide insights into how the relaxation processes present in FTS differ from those in MD, and could help explain why FTS can achieve such an efficient approach to equilibrium. It would also be insightful to compare the performance of a recent method for computing both *R*_g_ and contacts maps using FTS^57^ with more traditional approaches using MD simulations. Second, it would be interesting to compare FTS with MD using soft potentials other than the Gaussian potential considered in our work. Of particular interest is the dissipative particle dynamics (DPD) pair-potential^58,59^ which is widely used in soft particle simulations due to its short cutoff yet also amenable to FTS since the potential is positive-definite. Third, it would be beneficial to compare FTS to the many hybrid particle-field methods such as single-chain in mean field,^60,61^ theoretically informed coarse-grained,^62−64^ or theoretically informed Langevin dynamics.^17−20^ These hybrid methods can be considerably faster than MD due to very efficient methods to calculate the nonbonded interactions and the use of sophisticated Monte Carlo moves that relax the system through nonphysical pathways. It would be informative to see how the accuracy and performance of these hybrid methods compare to those of FTS across the different systems and conditions that we have considered in our work here.

While our primary objective in this work was to compare FTS and MD, an unexpected outcome of our work was the rediscovery of a very efficient method to compute the chemical potential of uncharged soft particle-based polymer models. While our method to compute the chemical potential in particle-based models (i.e., eq 32) has been present in the literature for over 20 years, it has generally been neglected in favor of more sophisticated methods like configuration bias Monte Carlo^37−39^ or the chain increment method.^40^ Our results demonstrate that eq 32 works remarkably well for uncharged soft polymer models and is accurate and efficient even for high densities and long polymers chains. It is also noteworthy that eq 32 is also straightforward to implement, parallelizes well on the GPU, and is easily incorporated into existing simulation codes. We anticipate that eq 32 could be useful in similar models with soft interactions such as dissipative particle dynamics^58,59^ or hybrid particle-field methods.^17−20,60−65^ Yet despite these benefits, our results clearly demonstrate that eq 32 is a less efficient route to the chemical potential than field-theoretic simulations, especially if Coulombic interactions are present.

A final implication of our work is for emerging methods^12,17^ that combine particle and field-theoretic simulations, especially the multi-representation simulations developed by our group.^21^ The strength of these methods relies on both the equivalence and asymmetry of particle and field-theoretic simulations. On one hand, these methods use the formal equivalence of particle and field-theoretic simulations to place them on a rigorous theoretical foundation that can be constructed without approximation. On the other, these methods leverage the asymmetric performance of particle and field-theoretic simulations to accelerate calculations by choosing the model representation where simulations will be most efficient. Our work here quantifies this equivalence and asymmetry for a broad range of different systems and opens up the possibility of multi-representation simulations involving any number of components and long-range Coulombic interactions.
